# On the Relative Importance of Li Bulk Diffusivity and Interface Morphology in Determining the Stripped Capacity of Metallic Anodes in Solid-State Batteries

**DOI:** 10.1021/acsenergylett.2c01793

**Published:** 2022-09-27

**Authors:** Marco Siniscalchi, Junliang Liu, Joshua S. Gibson, Stephen J. Turrell, Jack Aspinall, Robert S. Weatherup, Mauro Pasta, Susannah C. Speller, Chris R. M. Grovenor

**Affiliations:** † Department of Materials, University of Oxford, Oxford OX1 3PH, U.K.; ‡ The Faraday Institution, Didcot OX11 0RA, U.K.

## Abstract

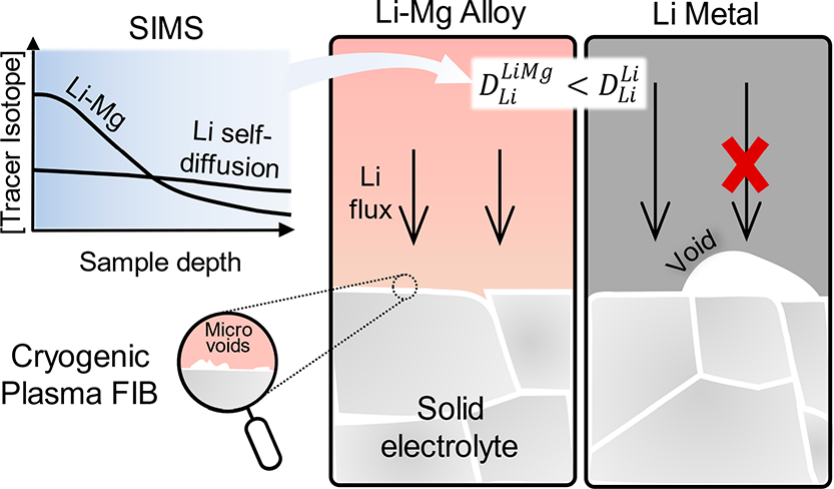

Lithium metal self-diffusion is too slow to sustain large current densities at the interface with a solid electrolyte, and the resulting formation of voids on stripping is a major limiting factor for the power density of solid-state cells. The enhanced morphological stability of some lithium alloy electrodes has prompted questions on the role of lithium diffusivity in these materials. Here, the lithium diffusivity in Li-Mg alloys is investigated by an isotope tracer method, revealing that the presence of magnesium slows down the diffusion of lithium. For large stripping currents the delithiation process is diffusion-limited, hence a lithium metal electrode yields a larger capacity than a Li-Mg electrode. However, at lower currents we explain the apparent contradiction that more lithium can be extracted from Li-Mg electrodes by showing that the alloy can maintain a more geometrically stable diffusion path to the solid electrolyte surface so that the effective lithium diffusivity is improved.

Lithium metal has recently regained popularity as an anode material for lithium-ion electrochemical cells owing to a specific capacity 10 times larger than offered by current graphite anodes (3860 mAh·g^–1^ versus 372 mAh·g^–1^).^1^ However, in cells where lithium metal is employed as the anode the bulk atomic diffusivity of lithium is often a limiting factor in determining the cycling behavior. In particular, with the advent of solid-state batteries where the lithium metal is paired with a solid electrolyte,^2^ fast bulk diffusivity of lithium atoms is needed to maintain a stable interface between the lithium metal electrode and the solid electrolyte under realistic cycling current densities.^3,4^ The mechanism for this is the migration of lithium ions into the solid electrolyte during stripping and the creation of vacancies at the surface of the lithium electrode, causing contact loss at the atomic scale if the diffusion of lithium atoms to the interface is not fast enough to refill the vacancies. These vacancies can then cluster to form voids, leading to macroscopic contact loss and to an increase in the electrode-solid electrolyte interface impedance.^5,6^ Eventually this can lead to the cell failure during subsequent cycles.^7^ External stack pressure can be applied to prevent contact loss, but it is unclear whether this would be practical at commercially relevant current densities. Therefore, in order to have a morphologically stable interface, rapid diffusion of lithium atoms to the interface is needed to avoid these local accumulations of vacancies, and it has been realized that it is important to understand the factors controlling this diffusion flux. In monatomic metals like lithium the bulk atomic diffusion process, also referred to as self-diffusion, consists of exchanging an atom with an adjacent vacancy, which, at the intermediate temperatures where batteries operate, is a monovacancy.^8^ This process has been previously investigated with nuclear magnetic resonance,^9−11^ tracer isotope mass spectrometry,^12^ and first-principles calculations.^8,13^ From these studies the lithium self-diffusion coefficient in lithium metal, *D*_Li_, is in the range of 10^–11^–10^–10^ cm^2^·s^–1^ at room temperature, with some discrepancies for different methods and experimental conditions, as summarized by Krauskopf et al.^14^ According to the defect relaxation model introduced by Schmalzried and Janek,^45^ in which *i*_crit_ ∝ , the critical stripping current for the formation of voids *i*_crit_ (at room temperature and zero external pressure) would be in the 50–200 μA·cm^–2^ range for such *D*_Li_ values.^3^

On the other hand, it has been reported that the use of lithium alloys can help to form a more stable interface with the solid electrolyte, and various lithium alloys have been used as anodes^14,15−18^ or as interlayers between lithium metal and solid electrolyte.^19−24^ The improved morphological stability of the interface has often been attributed to a fast lithium diffusivity in lithium alloys.^16,19,24,25^ During stripping, vacancies formed at the interface would quickly be refilled, while during plating the fast lithium diffusion into the bulk of the anode alloy would maintain the activity of lithium at the interface below one and limit the accumulation of lithium atoms at the interface. A number of alloys have been reported to have lithium diffusivities significantly exceeding lithium self-diffusivity, with chemical diffusion coefficients between 10^–8^ and 10^–6^ cm^2^·s^–1^ at room temperature measured by galvanostatic or potentiostatic electrochemical titration techniques in liquid electrolytes.^24,26−29^ Among these lithium alloys, the Li-Mg system has attracted particular interest.^15,30,31^ Mg has an exceptionally wide solubility range in Li, with the β-phase region spanning from 0 to 70 at.% Mg (see Figure 1a),^32^ so that there may be no phase transformation during electrochemical cycling of Li-Mg giving better microstructural stability.^14^ Magnesium is also a light element and Li-Mg alloys have a potential of ∼0 V versus Li/Li^+^, both desirable properties for optimizing the cell energy density.^33^ However, discrepancies in the values of lithium diffusivity in Li-Mg alloys can be found in the literature; diffusion coefficients around 10^–11^ cm^2^·s^–1^ have been obtained by nuclear magnetic resonance^34^ and neutron tomography,^35^ but values of 10^–8^–10^–7^ cm^2^·s^–1^ were obtained from potentiostatic electrochemical titration studies.^36^ A Li-Mg electrode with such fast lithium diffusivity would be able to sustain *i*_crit_ values 1 order of magnitude larger than those for a lithium metal electrode.^3^

**Figure 1:**
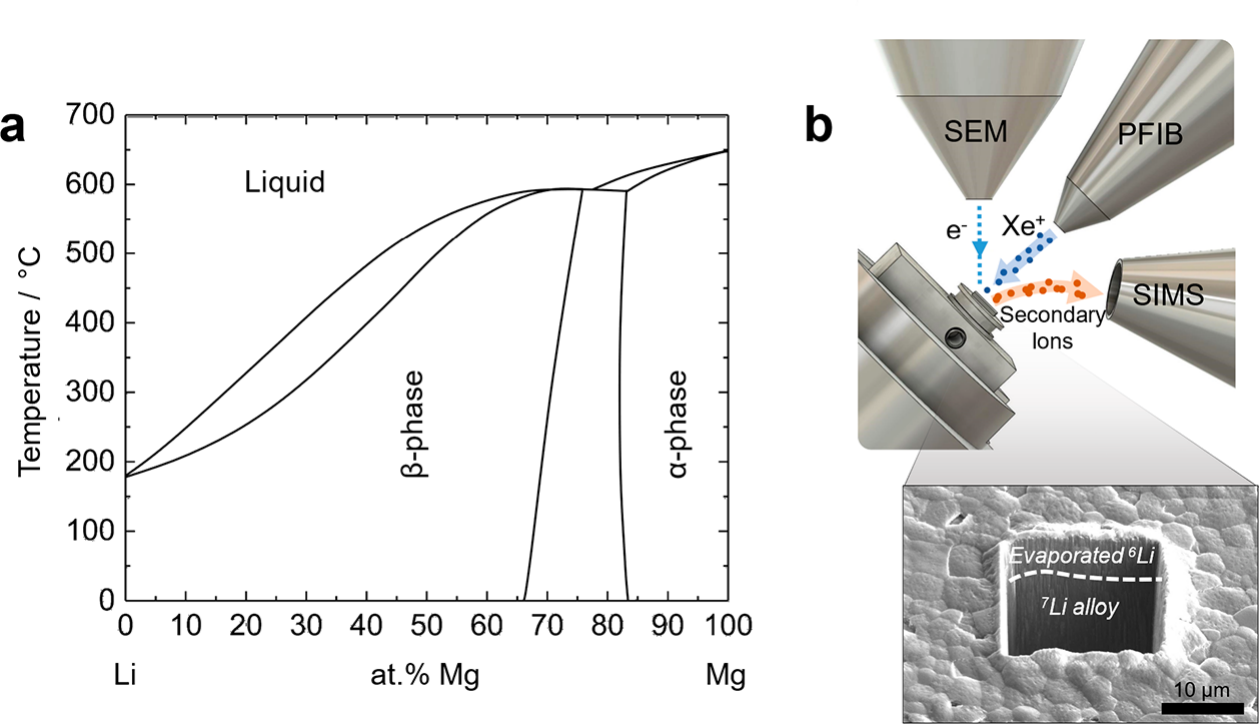
(a) Equilibrium phase diagram of the Li-Mg system.^32^ (b) Experimental setup for the measurement of the diffusivity with SIMS.

Given the importance of understanding the role of lithium diffusivity in controlling the morphology of the electrode-solid electrolyte interface, we aim here to resolve the inconsistency in the literature values by performing direct measurements of lithium diffusivity in elemental lithium and in Li-Mg alloys with up to 30 at.% Mg using an isotope tracer method. For this purpose, isotope heterostructures were prepared by coupling two materials with different stable isotope concentrations. To ensure intimate contact between the two materials, thermal evaporation was used to deposit a thin film of the tracer isotope onto bulk samples of different isotopic concentrations (experimental methods can be found in the Supporting Information). A secondary ion mass spectrometry (SIMS) detector fitted in a plasma focused ion beam (PFIB) instrument, as shown schematically in Figure 1b, was used to track the isotopic concentration as a function of depth, from which the diffusion coefficient can be derived. This direct measure of the tracer diffusive flux over the micron scale means that the diffusion coefficient obtained by SIMS is sensitive to the material microstructure (grain boundaries, dislocations, etc.) and is a good measure of the transport processes specifically in the anode metal, without needing to deconvolute the effect of electrode–electrolyte interfaces on Li^+^ transport. By contrast, methods such as nuclear magnetic resonance provide a measure of the random atomic-scale displacement over shorter distances and therefore a diffusion coefficient sensitive to one or few atomic jumps only. The SIMS method is also preferred over electrochemical techniques which only indirectly determine the chemical diffusion coefficient using a model containing several underlying assumptions that need to be satisfied to obtain accurate diffusion measurements.^37^

From our SIMS analysis we find that the lithium diffusivity in Li-Mg alloys agrees with the lower end of the literature values, and that it is about 1 order of magnitude slower than lithium self-diffusion in lithium metal. We thus suggest that any improvement in the performance of a solid-state battery with a Li-Mg alloy anode is not a result of a faster lithium diffusivity. In fact, employing a garnet solid electrolyte, we demonstrate that lithium metal outperforms the alloy anode for large stripping current densities. However, the alloy anode achieves a larger capacity at smaller stripping current densities and no external pressure thanks to a more stable morphological contact with the solid electrolyte as investigated by cryogenic PFIB sectioning.

## Lithium Diffusivity Measured by SIMS

**Figure 2:**
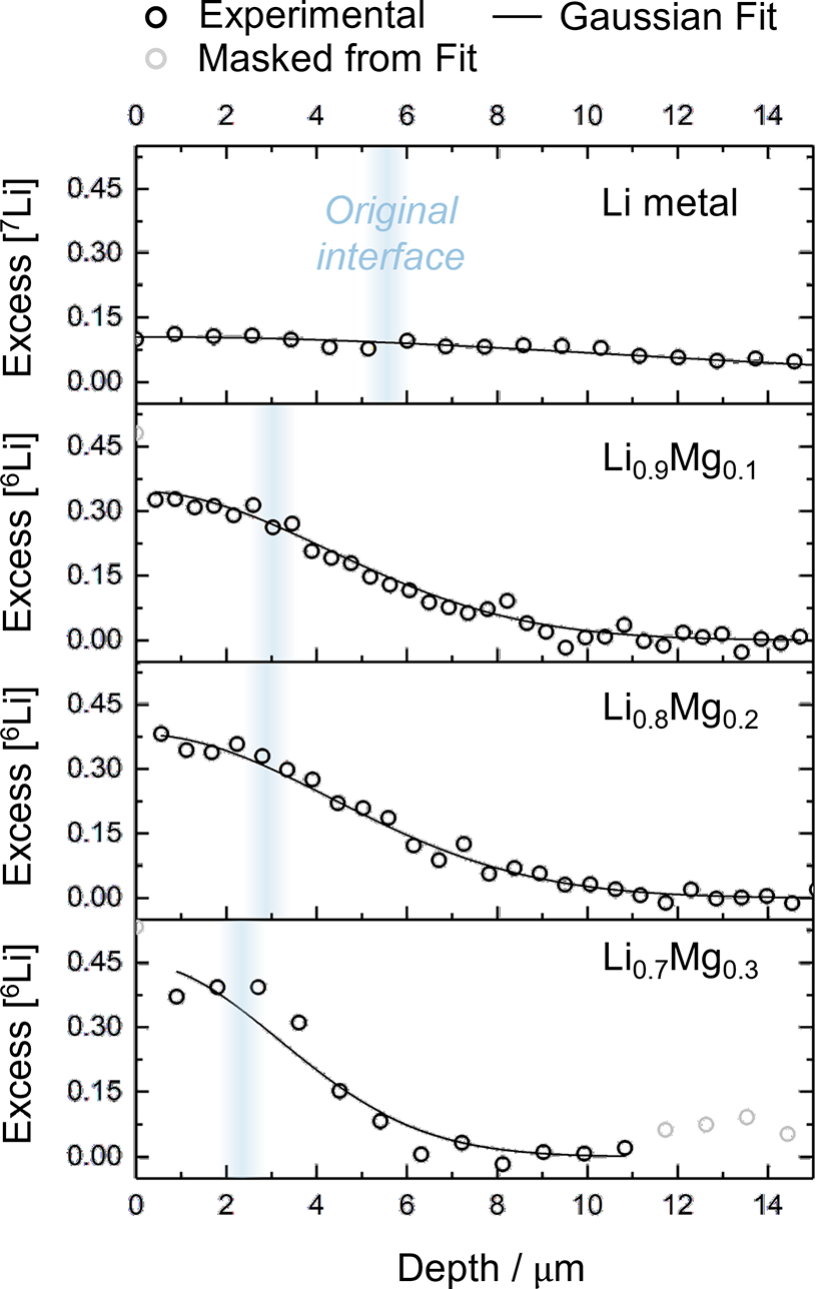
SIMS diffusion profiles of the excess concentration of the tracer isotope into the substrate taken 60 min from the start of the tracer deposition. From the top: ^7^Li into ^6^Li, then ^6^Li into ^7^Li_1–*x*_Mg_*x*_ with *x* = 0.1, 0.2, and 0.3. ○, experimental data; −, Gaussian fitting. The position of the original interface between the tracer and the substrate was inferred by cross sectional secondary electron imaging.

To investigate self-diffusion in lithium metal, a thin film of ^*7*^*Li* ∼5 μm thick was thermally evaporated onto ^*6*^*Li* (italics are used to distinguish lithium isotopes, ^7^Li and ^6^Li, from lithium metal with the natural isotopic abundance, ^*7*^*Li*, or from lithium metal isotopically enriched with ^6^Li, ^*6*^*Li*). To measure diffusion in ^7^Li-Mg alloys, ^*6*^*Li* was evaporated onto the alloy surface. Here we neglect the isotope effect, i.e. the possibility of ^6^Li diffusing faster than ^7^Li due to the difference in their masses, as the reported difference^11^ lies within the error bars of diffusion coefficients determined by most methods. Care was taken to perform all the operations in an inert Ar glovebox with <0.1 ppm values of O_2_ and H_2_O, and the surface contamination that can develop on lithium and Li-Mg substrates during storage was removed with a scalpel immediately prior to thermal evaporation. Naturally, as soon as the thermal evaporation process begins, interdiffusion of the two stable lithium isotopes also starts. A small increase in substrate temperature was detected during evaporation of the isotope thin film (as reported in the experimental methods in the Supporting Information), meaning the initial diffusion rate was slightly accelerated. Figure 2 shows typical diffusion profiles of the isotope tracer into different substrates taken 60 min from the start of the thermal evaporation. The excess concentration of the isotope tracer was fitted with the Gaussian solution of Fick’s second law (see experimental methods in the Supporting Information). The first data points can lie at an artificially high value due to the effect of surface contamination on the sputter yield, and these points were removed from the Gaussian fitting.

The diffusion profile of ^7^Li into ^*6*^*Li* in Figure 2 results in the calculation of a lithium self-diffusion (tracer diffusion) coefficient, *D*_Li_*, of (1.6 ± 0.1) × 10^–10^ cm^2^·s^–1^, which is in reasonable agreement with previous literature reports.^12^ However, the lithium diffusivity is not improved in the Li-Mg alloys, which have measured intrinsic diffusion coefficients for Li, *D*_Li_, of (2.4 ± 0.1), (2.6 ± 0.1), and (1.4 ± 0.2) × 10^–11^ cm^2^·s^–1^ for 10, 20, and 30 at.% Mg alloys, respectively, based on the diffusion profiles in Figure 2. This slower diffusivity can also be seen in Figure 2 by the fact that the surface concentration of the tracer isotope on the Li-Mg alloys is greater in all cases than on the elemental lithium metal. Additional SIMS diffusion profiles for Li-Mg alloys taken at different times after the start of the tracer deposition are reported in Figure S1 in the Supporting Information.

To interpret this data, we note that the β-phase in Li-Mg alloys is a substitutional solid solution, and since the Goldschmidt radii of Li and Mg differ by only a few percent we may assume that lithium diffuses in Li-Mg by the same vacancy mechanism as in elemental lithium. Another possible difference between the lithium metal and Li-Mg alloy materials that might influence the measured diffusivity is the grain size, but as shown in Figure S2 this is of the order of 100 μm in all materials, and so the grain boundary contribution to the overall diffusion fluxes should be both similar and negligible. We note here that when considering a lithium flux diffusing into a Li-Mg alloy there is a compositional gradient that should introduce an associated activity gradient (hence the use of the label intrinsic diffusion coefficient, *D*_Li_), but that in general adding a higher melting point element, B, to a lower melting point matrix, A, should decrease the value of *D*_A_.

The *D*_Li_ values agree with the more conservative literature values on Li-Mg alloys, such as Korblein et al. (<7 × 10^–11^ cm^2^·s^–1^ using nuclear magnetic resonance)^34^ and Zhang et al. (6 × 10^–11^ cm^2^·s^–1^ using neutron tomography),^35^ as well as with the study on Li-Mg in contact with a solid electrolyte by Krauskopf et al. (3 × 10^–11^ cm^2^·s^–1^).^14^ The discrepancy with the literature showing faster lithium diffusivity in Li-Mg alloys obtained by potentiostatic electrochemical titration might be explained by the fact that the diffusion coefficients obtained by electrochemical methods are strongly influenced by the microstructure of the alloys used and their surface morphology, with larger diffusion coefficients for more porous structures (like the ones studied by Gole et al.).^29,36^ In fact, for such methods the knowledge of the true alloy surface area is needed to calculate the diffusion coefficient.^38^ This and other sources of uncertainties can alter the diffusivity values measured by electrochemical titration methods as much as 4 orders of magnitude,^37^ while tracer diffusion methods like the one employed here directly probe the bulk diffusivity without the need for model assumptions.

## Implications for Solid-State Batteries

**Figure 3:**
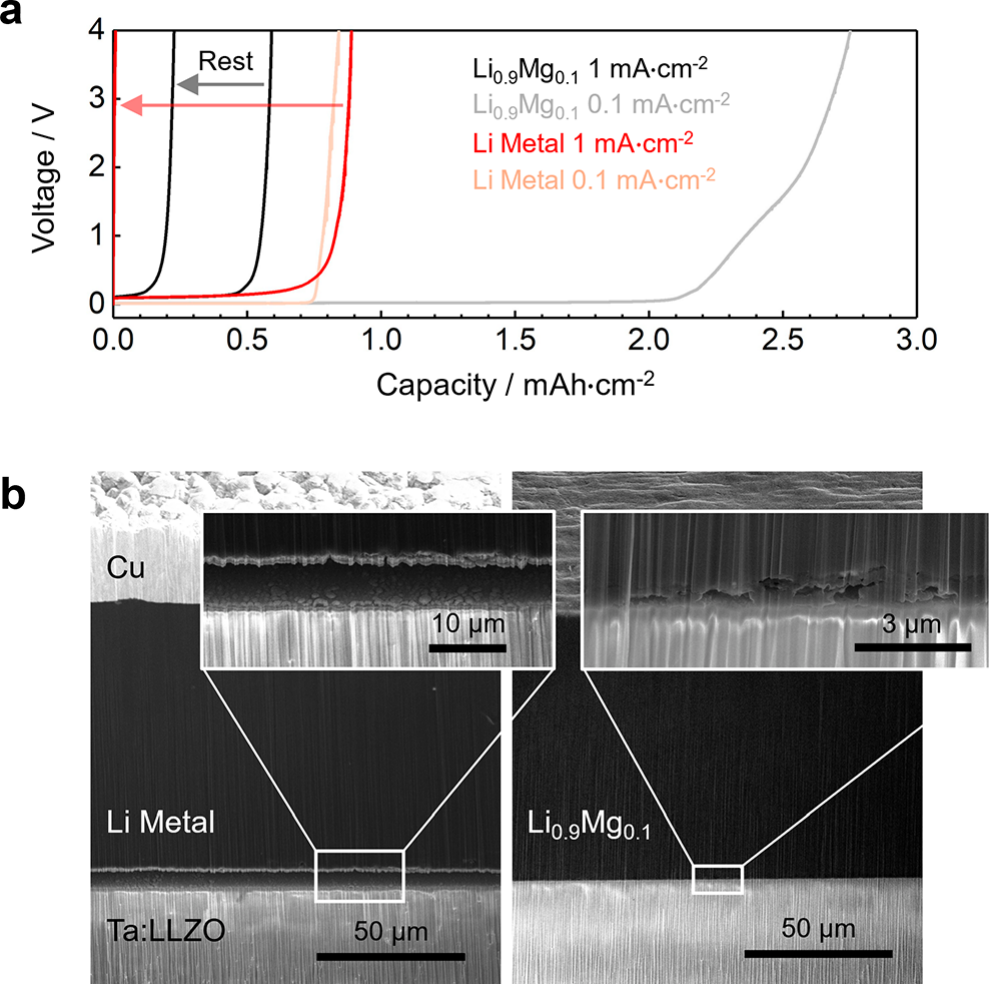
(a) Stripping experiments for lithium and Li_0.9_Mg_0.1_ electrodes at 1 mA·cm^–2^ and 0.1 mA·cm^–2^. The cells were cycled at 30 °C and without external pressure. The counter electrode was lithium metal. For 1 mA·cm^–2^, after the cutoff voltage of 4 V was reached the cell was rested for 2 h, then stripping was repeated. (b) Secondary electron images of cryogenic PFIB cross sections of the working electrodes after the first stripping step at 1 mA·cm^–2^. A large, continuous gap is formed between lithium and Ta:LLZO, while a more localized porosity is present in the Li_0.9_Mg_0.1_ electrode.

In the context of the performance of solid-state batteries, the lithium self-diffusivity *D*_Li_* found in this study would be predicted from the model of Schmalzried and Janek^45^ to be too low for a lithium electrode to maintain a morphologically stable interface with a solid electrolyte even at small current densities (50–200 μA·cm^–2^), so that voids will form during stripping unless a large external pressure is applied.^3^ The smaller *D*_Li_ values in the Li-Mg alloys would suggest even slower lithiation and delithiation kinetics in these alloy anodes. We have confirmed this by performing stripping experiments using lithium and Li_0.9_Mg_0.1_ electrodes in contact with a lithium garnet solid electrolyte Ta:LLZO. No external pressure was applied during stripping, i.e. the lithium flux in the anode depends only on the lithium diffusivity and the effect of creep deformation can be ruled out. The starting interfacial impedance of the two-electrode solid-state cells was consistently low thanks to the Ta:LLZO surface treatment used in this study (Figure S3 and experimental methods in the Supporting Information). Figure 3a reports the potential profile during delithiation at a current density of 1 mA·cm^–2^. The polarization of the cell increases with time as the lithium concentration at the solid electrolyte surface decreases. Cross sectioning these interfaces by cryogenic PFIB (Figure 3b and Figure S4 in the Supporting Information) shows that for the lithium metal electrode this can be explained by the formation of large voids and an almost complete loss of contact, whereas the presence of an element which is not stripped, i.e. magnesium, encourages the preservation of a more stable contact morphology between the Li_0.9_Mg_0.1_ alloy electrode and the solid electrolyte. Lithium is stripped from both electrodes at the same rate and, even though large voids are largely prevented by the use of Li_0.9_Mg_0.1_, its slower bulk lithium diffusivity (*D*_Li_) restricts the capacity extracted from the alloy to a smaller value. The cycling performance is verified using at least two cells of each type (Figure S4). Interestingly, if the Li_0.9_Mg_0.1_ electrode is rested for a few hours after the first delithiation, lithium can diffuse back to the solid electrolyte surface due to the maintained diffusion path and some additional capacity can be extracted in a subsequent stripping step (Figure 3a). By contrast, the complete loss of contact between the lithium metal electrode and the solid electrolyte, which is not recovered in the absence of external pressure, prevents any further stripping even after a rest period.

In Figure 3a the potential profiles at 0.1 mA·cm^–2^ are also reported. When a smaller stripping current is used a similar capacity is achieved by the lithium metal electrode, suggesting that a similar number of voids are formed after the same stripped capacity if no external pressure is applied. The capacity extracted from the Li_0.9_Mg_0.1_ electrode, however, is more than 4 times higher than the one at 1 mA·cm^–2^ as a consequence of the ability of Li_0.9_Mg_0.1_ to retain good contact with the solid electrolyte. This result suggests that if the delithiation rate is low enough that the bulk diffusion of lithium atoms can supply a sufficient flux to the interface, it is the morphological stability of the interfacial contact that plays a more important role in determining the final capacity. Li-Mg electrodes with 20 and 30 at.% Mg were not used here as they have higher hardness, making it more difficult to assemble solid-state cells, and lower energy density. However, all the Li-Mg alloys reported above have comparable diffusion coefficients, suggesting a similar delithiation behavior in contact with Ta:LLZO.

It should be kept in mind that in real operating conditions the alloy electrode would see a variation in the lithium activity which is difficult to predict, and which might alter the lithium diffusivity (*D*_Li_ should depend on the local concentration of Li) and the charge transfer efficiency.^14,39^ However, from the results reported above and from previous studies^14,34,36^ we expect the diffusion coefficient to decrease with increasing magnesium content, which is consistent with the steep increase of the melting temperature (see Figure 1a). Nevertheless, Li-Mg alloys seem to be able to provide the lithium atoms in the bulk of the anode continuous access to the solid electrolyte surface through the partly delithiated Li-Mg phase, while clearly no lithium diffusion can occur through a void in the lithium electrode. Therefore, the behavior of Li-Mg alloys can be understood by considering an *effective* lithium diffusivity that takes into account the bulk lithium diffusivity *and* the way in which the different void morphologies control the contact area between the electrode and the solid-electrolyte. When voids are formed in a lithium metal electrode, its effective diffusivity rapidly decreases. A Li-Mg electrode has a smaller initial effective diffusivity, but the interface morphology is more stable over cycling thus enhancing the capacity at small stripping rates.

## Interface Contamination

**Figure 4:**
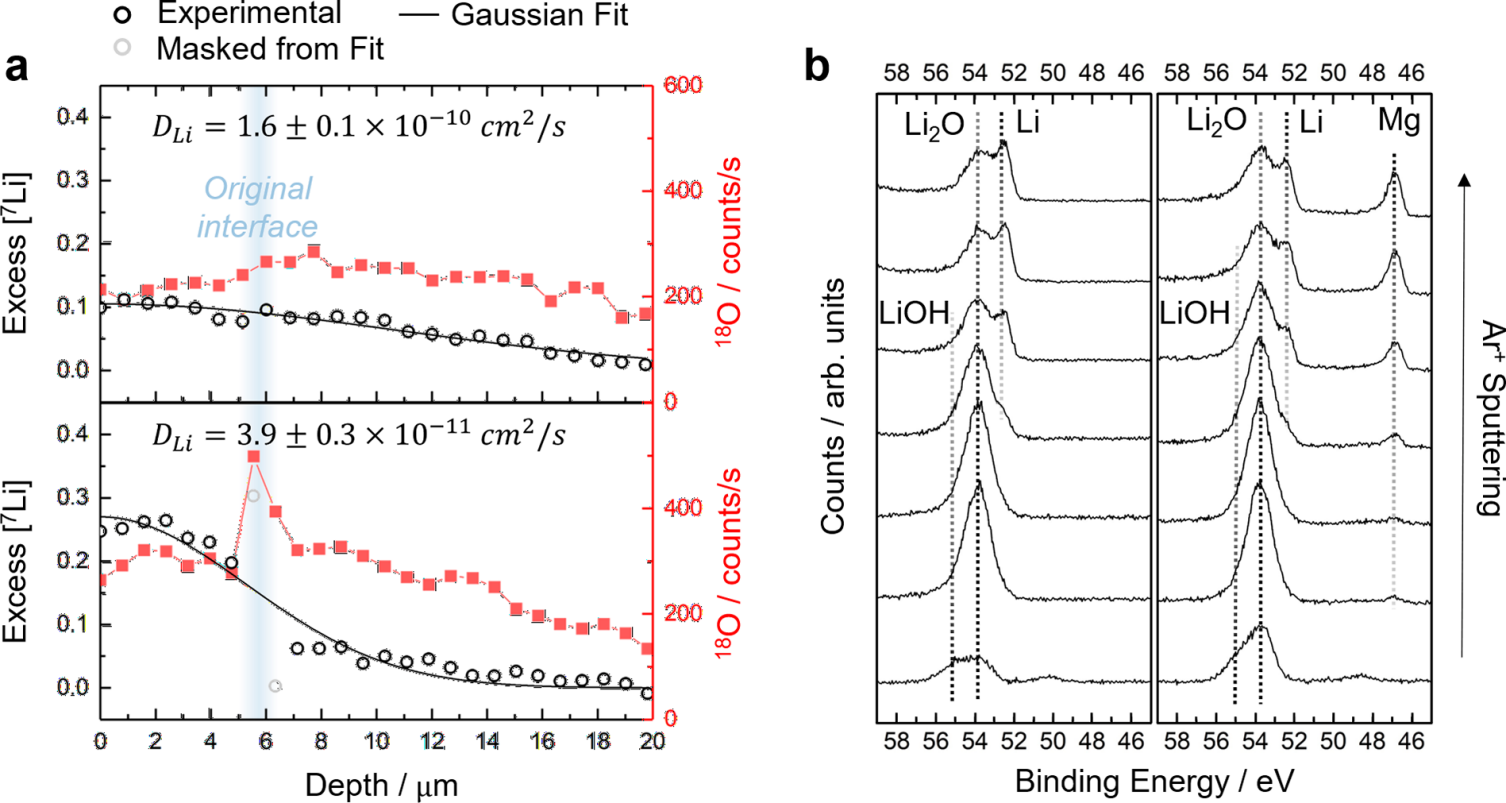
(a) Diffusion profile of ^7^Li into ^6^Li including the ^18^O SIMS signal at 60 min after the start of the tracer deposition. Top panel: ^6^Li substrate prepared in a glovebox atmosphere with <0.1 ppm of H_2_O and O_2_ (same profile as in Figure 2). Bottom panel: glovebox atmosphere with ∼1 ppm of H_2_O and O_2_. (b) XPS signal of the Li 1s and Mg 2p regions for fresh surfaces of ^6^Li (left) and ^7^Li_0.9_Mg_0.1_ (right). Ar^+^ ions were used for depth profiling with the following sputtering steps: 2 × 2 min, 2 × 5 min, and 2 × 15 min.

During the lithium diffusivity experiments, care was taken to minimize the presence of contamination at the interface between the evaporated tracer thin films and the ^*6*^*Li* or ^7^Li-Mg substrates. Lithium and Li-Mg alloy samples will rapidly react with trace moisture, oxygen, carbon dioxide, and nitrogen in a glovebox and even in the vacuum in the thermal evaporation chamber.^40,41^ Mindful of this, exposure of the fresh lithium and Li-Mg alloy surfaces to the glovebox atmosphere was kept to a minimum (<1 min) before evacuation of the thermal evaporation chamber. However, the presence of some contamination is unavoidable, and in order to understand its effect on the lithium diffusivity, Figure 4a compares two diffusion profiles of ^7^Li into ^*6*^*Li* where the ^*6*^*Li* substrate was prepared in glovebox atmospheres with different contamination levels prior to the ^*7*^*Li* tracer deposition. For a glovebox atmosphere with ∼1 ppm of H_2_O and O_2_, the ^18^O signal collected with SIMS shows a substantial peak where the original ^*7*^*Li*–^*6*^*Li* interface was, which is direct evidence of contamination of the ^*6*^*Li* surface in the less well controlled environment. Oxygen is known to strongly enhance the ionization probability of electropositive elements,^42^ so the excess [^7^Li] data points at the interface do not follow the diffusion profile and were removed from the Gaussian fitting. In this case the measured diffusion coefficient is 1 order of magnitude lower than the value previously reported, so even a small amount of contamination can have a big impact on the measured lithium diffusivity. The good agreement between our results and most of the literature data on direct measurements of lithium diffusivity discussed above suggests that the amount of contamination under well-controlled glovebox conditions does not strongly distort the lithium interdiffusion measurements.

We have also studied the levels of surface contamination on ^*6*^*Li* and Li-Mg surfaces by XPS analysis (Figure 4b). Even fresh surfaces contain some signals from lithium oxide and hydroxide, but in situ Ar^+^ sputtering in the XPS chamber can reveal the underlying metallic signal,^43^ which is exposed after the same duration of Ar^+^ sputtering for all samples. The same contaminant species are seen across all surfaces (see Figure S5 for the full XPS data set). From this analysis we conclude that the influence of unavoidable surface contamination on the measurements of lithium diffusivity should be similar on both lithium metal and Li-Mg. It would be interesting to study the effect of more severe lithium surface contamination (as would be expected for prolonged exposure to glovebox conditions or for a less well controlled environment) on the apparent lithium diffusivity, and this will be the subject of a following study.

In summary, the diffusivity of lithium in Li-Mg alloys with 10, 20, and 30 at.% Mg as well as lithium self-diffusivity were studied with an isotope tracer method, employing SIMS to track the isotope diffusion. In contrast to some reports in the literature, our results show that the lithium diffusivity in Li-Mg is substantially slower than lithium self-diffusion: ∼2 × 10^–11^ versus 1.6 × 10^–10^ cm^2^·s^–1^. This is important information for the design of future solid-state batteries. To date, the stable electrode–electrolyte interface and good cycling performance of cells made with a wide range of lithium alloys have often been attributed to faster lithium diffusivity. Our experiments show that this suggestion must be challenged, because despite the smaller lithium bulk diffusion rate a Li-Mg alloy can provide a much larger stripping capacity than a lithium metal electrode if a relatively small current density is used. We explain this behavior by revealing that the Li-Mg alloy maintains a stable interfacial contact with the solid electrolyte so that lithium atoms can continuously diffuse to the electrolyte surface; i.e., it is the improved morphology of the interface that provides the operational stability. By contrast, the large voids in the lithium metal electrode block the transport of lithium atoms even when the bulk diffusion coefficient in the anode is larger. However, when larger current densities are used, the slower bulk diffusion kinetics in the Li-Mg alloy electrode ultimately limit the stripping capacity so that under these conditions a lithium metal electrode performs better. Hence, if solid-state batteries are to be used at commercially relevant current densities the search for a lithium alloy with a fast lithium bulk diffusivity is still important, not only for the obvious influence during lithium stripping, but also to relieve interfacial stresses^44^ and to keep the lithium activity at the interface <1 during lithium plating. To do so, we believe that isotope tracer methods may be useful to probe the bulk diffusivity directly, without the need for model assumptions. Finally, a small amount of unavoidable surface contamination from the glovebox atmosphere does not seem to suppress the diffusion of lithium, but the effect of more serious surface reaction still needs to be fully understood.

## Supporting Information Available

Experimental methods; additional SIMS diffusion profiles for Li-Mg alloys; XRD, XPS, and SEM images of lithium and Li-Mg alloys; EIS of the Ta:LLZO solid-state cells; typical positive ion mass spectrum collected by SIMS (PDF)
