# Supplementary material for: On the Relative Importance of Li Bulk Diffusivity and Interface Morphology in Determining the Stripped Capacity of Metallic Anodes in Solid-State Batteries
Source: ACS Energy Lett. 2022 Sep 27;7(10):3593–9. doi: 10.1021/acsenergylett.2c01793 (PMC9578048; doi:10.1021/acsenergylett.2c01793)
Supplement: Supplementary file 1 — nz2c01793_si_001.pdf [file nz2c01793_si_001.pdf]

On the relative importance of Li bulk diffusivity and interface morphology in determining the stripped capacity of metallic anodes in solid-state batteries

Marco Siniscalchi,[†,‡] *Junliang Liu,*[†] *Joshua S. Gibson,*[†,‡] *Stephen J. Turrell,*[†,‡] *Jack Aspinall,*[†,‡] *Robert S. Weatherup,*[†,‡] *Mauro Pasta,*[†,‡] *Susannah C. Speller,*[†] *Chris R. M. Grovenor**[,†,‡]

[†]Department of Materials, University of Oxford, Oxford, OX1 3PH, UK

[‡]The Faraday Institution, Didcot, OX11 0RA, UK

*chris.grovenor@materials.ox.ac.uk

Supporting Information

Experimental methods

Lithium metal (foil, 99.9%, Alfa Aesar) and magnesium metal (turnings, 99.98%, Alfa Aesar) were used for the casting of the Li-Mg alloys in a tube furnace (MTI KSL-1200X-J-UL) within an argon-filled glovebox (O_2 and H_2O <0.1 ppm). Lithium metal was heated to 100 °C above the liquidus temperature in a custom-made stainless-steel crucible, lined with stainless-steel

foil. For 20 and 30 at. % Mg samples, molybdenum foil was used instead, as the Li-Mg melt was observed to react with the steel above 450 °C. The molten lithium was removed from the furnace once at temperature, any slag carefully removed with a stainless-steel spatula, and an appropriate mass of magnesium turnings were fully dissolved in the molten lithium. The crucible was then returned to the furnace for 2 hours before slowly cooling to room temperature. Inductively coupled plasma optical emission spectroscopy (ICP-OES) analysis (PerkinElmer Optima 8000) was then used to confirm the Mg content as 10, 21, and 31 at. %.

^{6}Li-enriched lithium metal (chunks, 95 at. % ^{6}Li, Sigma Aldrich) and lithium metal granules with a natural isotopic ratio (99%, Sigma Aldrich) were used for thermal evaporation, and in the article these are referred to as ^{6}Li and ^{7}Li respectively to distinguish them from the pure isotopes. The isotopic content of as-received lithium metals was tested by SIMS. The measured concentration of ^{7}Li in the lithium metal granules is 91% in reasonable agreement with the natural isotopic abundance of 92.5%; the concentration of ^{6}Li in the ^{6}Li-enriched material is 95.1 % as specified by the supplier. The deposition of lithium thin films was carried out with a thermal evaporator (MB EVAP, MBraun) built within an argon-filled glovebox (MBraun, O_2 and H_2O <0.1 ppm). The substrate was a freshly cut surface of either ^{6}Li or ^{7}Li-Mg, gently scratched with a stainless steel blade just before it was mounted in the thermal evaporation chamber, ~20 cm from the evaporation source. The evaporation chamber was immediately evacuated down to a background pressure lower than 1×10^{-5} mbar before starting the deposition process, and a quartz crystal microbalance was used to monitor the deposition rate (25-30 Å s^{-1}) until a thickness of ~3-5 μm was obtained. Thinner films would diffuse too quickly into the substrate, even at room temperature, and the concentration profile would be lost, while thicker films would take a long time to deposit, risking excessive thermal heating during evaporation, and would require very protracted SIMS depth profiling. The temperature of the substrates was monitored by a thermocouple in contact with the substrate surface. The temperature slowly increased from 26 °C (glovebox atmosphere) to a maximum of 35 °C at the end of the evaporation (15-20 minutes), at a similar rate for all the substrates used in this work.

After thermal evaporation the samples were transferred to the PFIB (Helios G4 PFIB CXe DualBeam, Thermo Scientific) in an argon-filled transfer vessel (Gatan iLoad). A 30 kV accelerating voltage and a 4 nA current were used for the Xe^{+} beam. These parameters were calibrated to ensure a fast removal of material with a minimal beam damage. During PFIB

sputtering, a crater evolves at the sample surface and the secondary ion intensities of the two Li isotopes, $^{6}Li^{+}$ and $^{7}Li^{+}$, were recorded by SIMS (Hiden Analytical EQS) at each PFIB slice, so that a profile as a function of the sputtering time was obtained. The sample was held at room temperature throughout, so inter-diffusion of the thin film and bulk substrate occurred throughout the experiment. The sputtering area was 20 × 20 μm^2, but only about 20% of this area was used for the analysis of the diffusion profile in order to exclude crater edge effects. At the end of each experiment, the craters were examined by secondary electron (SE) images to convert the sputtering time to a sputtering rate.

The $^{6}Li^{+}$ and $^{7}Li^{+}$ secondary ion intensities were corrected to account for the formation of LiH and LiH_2^{+} species produced by the reaction of sputtered Li^{+} with trace hydrogen that is present in the PFIB chamber even in the ultra-high vacuum conditions used for SIMS analysis (see Figure S6). The diffusion profiles were then fitted with the "thin film" Gaussian solution of Fick's 2nd law $C(x,t) = \frac{N}{\sqrt{\pi D t}} e^{-x^2/4D_{Li}t}$ where $C(x,t)$ is the excess lithium isotope concentration in the sample with respect to the background concentration, $N = \int_0^\infty c(x,t)dx$ is the total amount of the diffusing species, and D_{Li} is the diffusion coefficient.[1] This solution is suitable for isotope diffusion experiments with a fixed amount of diffusing species, i.e. a thin film, where the effects of short circuit diffusion through grain boundaries can be ignored because of the very large grain size in bulk metallic Li and Li-Mg samples (Figure S2c).[2]

A Zeiss Merlin SEM with an argon-filled transfer vessel (Gatan iLoad) was used for imaging of the grain size of the substrates used in this study. XRD (Empyrean, Malvern Panalytical) was carried out to analyse their phase purity with the samples protected from the atmosphere by polyimide tape (Figure S1). The surfaces of lithium metal and of Li-Mg alloys were characterised by X-ray photoelectron spectroscopy (XPS) using an ULVAC Phi Versaprobe III XPS with a water-cooled monochromatic Al Kα source (hν 1486.6 eV, 15 kV anode voltage, 25 W beam power). The samples were prepared in an argon-filled glovebox (O_2 and H_2O <0.1 ppm). Freshly cut surfaces were gently scraped with a stainless steel blade just before transferring the samples to the XPS in an argon-filled transfer vessel (ULVAC Phi GmbH, <1 minute from the glovebox to the XPS chamber). The samples were electrically connected to the sample holder with conductive carbon tape. Ar^{+} ion beam sputtering was used for depth profiling (4 kV acceleration voltage, 3x3 mm^2 raster, 2.8 μA beam current). The XPS data were energy calibrated relative to the O 1s lithium oxide signal at 528.5 eV. Core level spectra were collected with a pass energy of 55 eV and a step size of 0.05 eV/step. The analysis

chamber pressure was maintained below 5×10^{-9} mbar during measurement. Data analysis was performed using the CasaXPS software package.

For the solid-state cell preparation, $Li_{6.4}La_3Zr_{1.4}Ta_{0.6}O_{12}$ powder (99.9%, Ampcera) was cold-pressed in a graphite die and then spark-plasma sintered (Dr Fritsch DSP 507) into dense pellets at 1200 °C and 50 MPa for 5 minutes. The pellets were dry polished in the glovebox down to 1 µm with a diamond lapping film until the surface was visually shiny. A rapid acid treatment[3] of 10 seconds in a 1M HCl water solution was employed to remove the surface Li_2CO_3 contamination from the Ta:LLZO pellets. Then the pellets were immediately brought back to the glovebox and 2-electrode cells were assembled with 200 µm thick Li or $Li_{0.9}Mg_{0.1}$ electrodes, which were gently pressed onto the solid electrolyte after applying 2 mm Ø Kapton tape masks to ensure a constant electrode area during cycling. Then a thermal preconditioning step was carried out by leaving the cells at 170 °C for 2 hours with ~2 MPa of pressure to facilitate the formation of a good interface between the electrodes and the solid electrolyte.[4] The cells were sealed in Mylar pouch bags with Cu current collectors and removed from the glovebox for testing. Potentiostatic electrochemical impedance spectroscopy (MTZ-35, BioLogic) was performed in a frequency range of 3.7 MHz – 1 Hz with a 10 mV perturbation, to ensure that the starting interfacial impedance of the cell was small. The impedance spectra were fitted using an equivalent circuit model in the ZView software package. Galvanostatic cycling (VMP3, BioLogic) was performed at 30 °C within a temperature control chamber and without external pressure. After cycling, the cells were carefully disassembled and transferred to the same PFIB instrument used for the diffusivity experiment fitted with a cryogenic stage. Cross-sectioning of the Ta:LLZO cells was performed at -150 °C to avoid beam damage and melting of the lithium or Li-Mg electrode. Secondary electron imaging was carried out with a 10 kV and 0.1 nA electron beam.

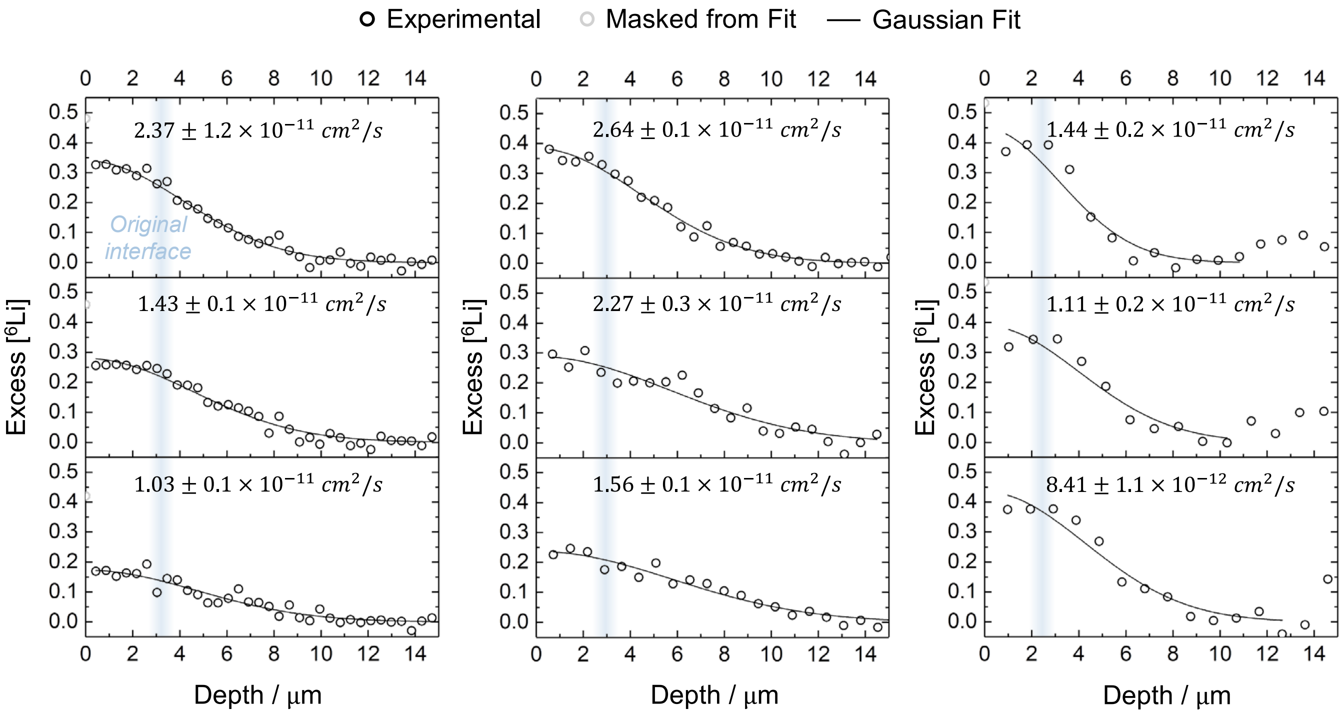

Figure S1. SIMS diffusion profiles of the excess concentration of the ^{6}Li isotope into $^{7}Li_{1-x}Mg_{x}$ with x = 0.1, 0.2, and 0.3 (panels from left to right). The profiles were taken on each sample 60, 120, and 180 minutes from the start of the tracer deposition (panels from top to bottom). The lithium diffusion coefficient D_{Li} obtained from the Gaussian fitting is reported above each diffusion profile. There is reasonable agreement between the diffusion coefficients measured from the same sample. The position of the original interface between the tracer and the substrate was inferred by cross sectional secondary electron imaging.

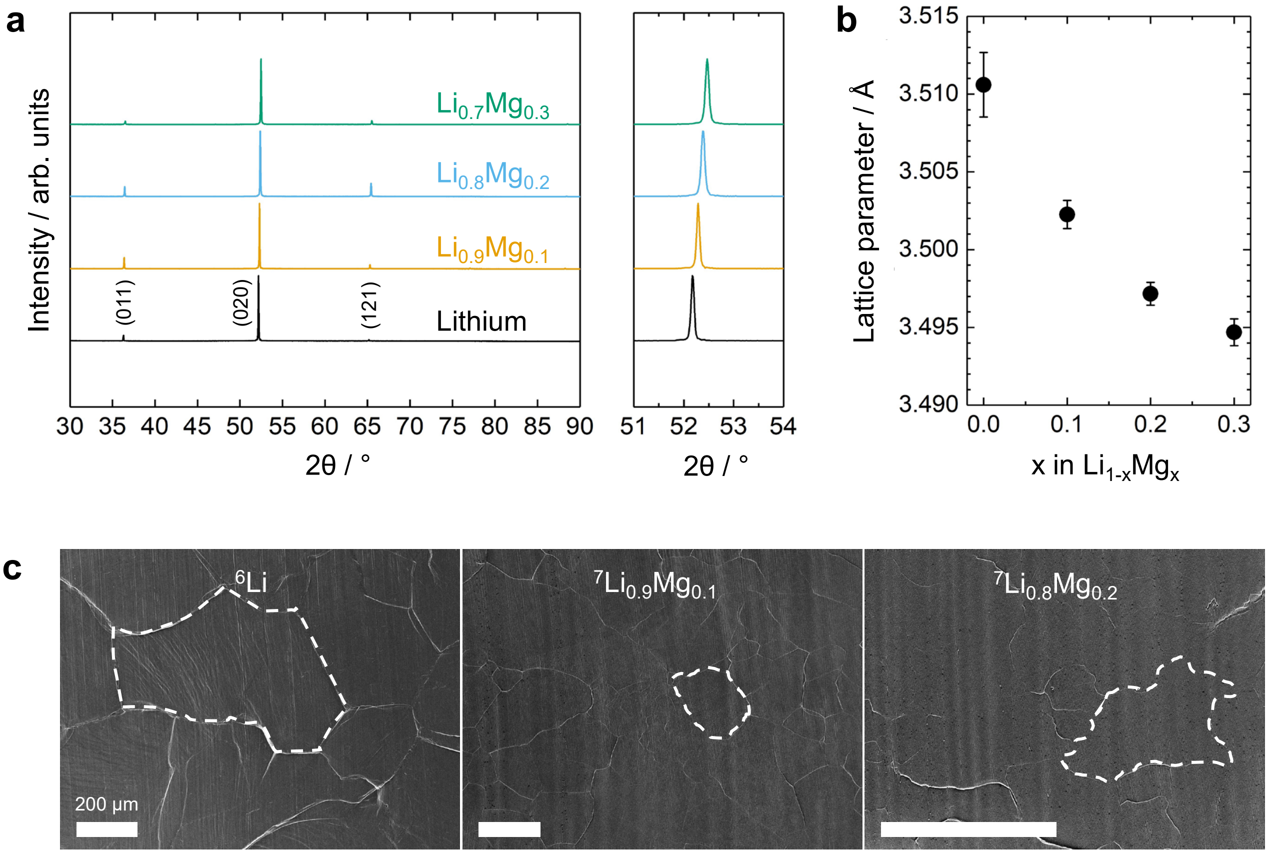

Figure S2. a) X-ray diffraction patterns of lithium metal and of the Li-Mg alloys. b) Lattice parameter as a function of magnesium content. c) SEM images of the substrates employed in this study. The grain size is at least tens of μm for all the samples, so that the contribution from grain boundary diffusion to the total diffusivity is negligible. The $Li_{0.7}Mg_{0.3}$ alloy is not included here as it was more difficult to manipulate.

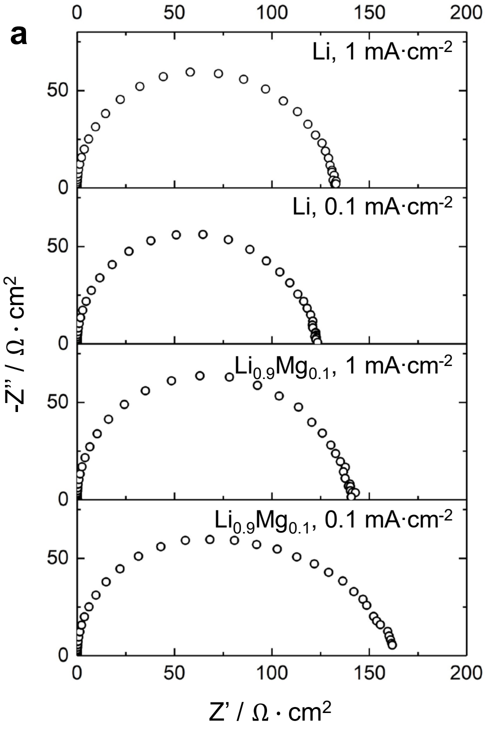

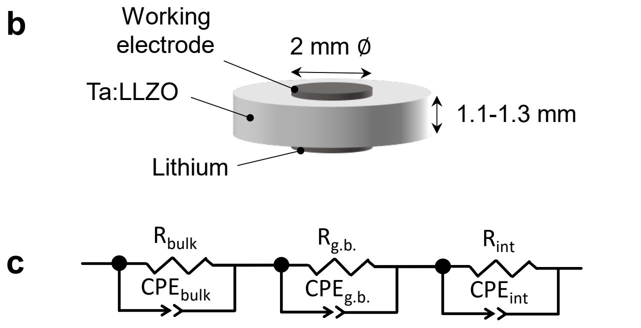

Working electrode, *i*	R_{bulk}	$R_{g.b}$	R_{int}
Li, 1 mA·cm^{-2}	69.9 ± 9.6	55.0 ± 7.2	4.3 ± 2.4
Li, 0.1 mA·cm^{-2}	65.1 ± 1.0	52.5 ± 1.1	2.7 ± 0.6
$Li_{0.9}Mg_{0.1}$, 1 mA·cm^{-2}	67.5 ± 9.6	55.0 ± 7.2	4.9 ± 1.8
$Li_{0.9}Mg_{0.1}$, 0.1 mA·cm^{-2}	83.0 ± 3.0	60.0 ± 4.3	10.8 ± 2.5

Values are in Ω · cm^2

Figure S3. a) Electrochemical impedance spectroscopy performed on the solid-state cells prior to the stripping experiments in Figure 3a in the main text, labelled as: working electrode, current density. b) Two-electrode cell schematics where the working electrode is either pure lithium metal or a Li-Mg alloy. c) Equivalent circuit model for the fitting of electrochemical impedance spectra and table with bulk, grain boundary, and interfacial resistance (single interface) values obtained by the fitting. The starting interfacial resistance values are very small thanks to the surface treatment of the Ta:LLZO solid electrolyte pellets.

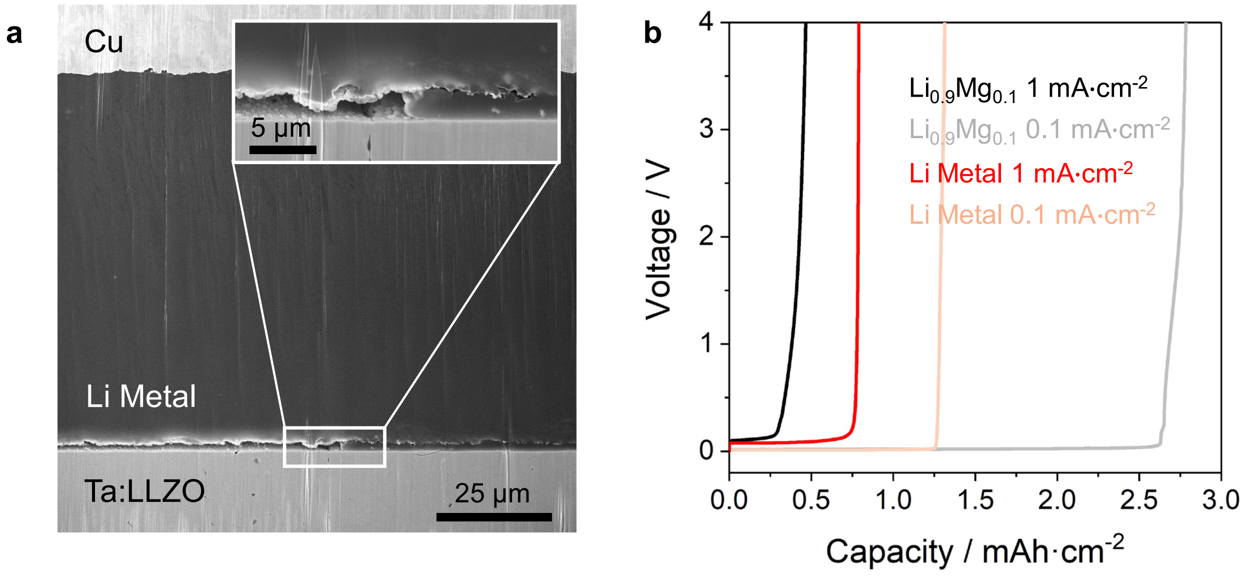

Figure S4. a) Additional secondary electron image of a cryogenic PFIB cross section of lithium metal working electrodes after stripping to 4 V at 1 mA·cm^{-2}. The inset shows that the Li/Ta:LLZO interface is maintained in some regions. b) Replicate stripping experiments for lithium and $Li_{0.9}Mg_{0.1}$ electrodes at 1 mA·cm^{-2} and 0.1 mA·cm^{-2}. The cells were cycled at 30 °C and without external pressure. The counter electrode was lithium metal.

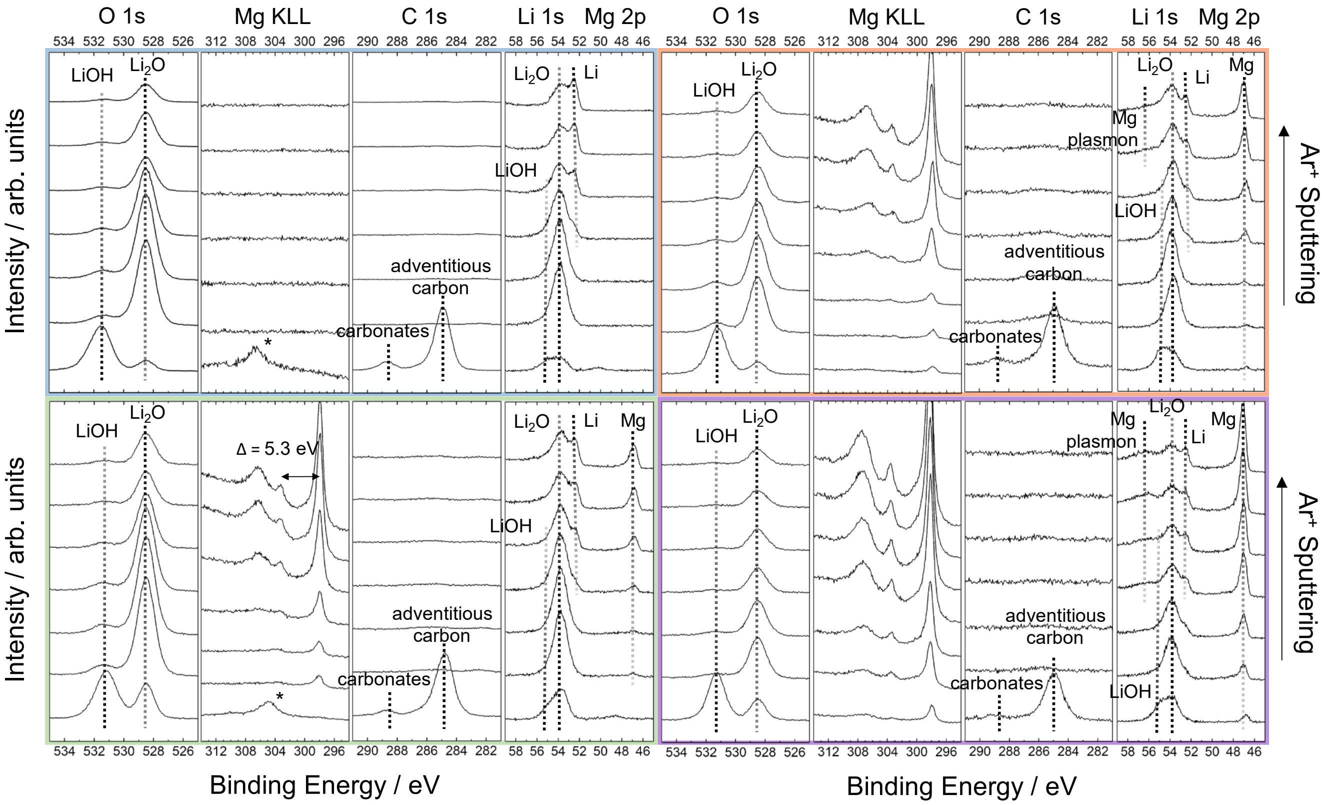

Figure S5. Full XPS data set for lithium metal (top left), $Li_{0.9}Mg_{0.1}$ (bottom left), $Li_{0.8}Mg_{0.2}$ (top right), and $Li_{0.7}Mg_{0.3}$ (bottom right). Ar^+ ions with an accelerating voltage of 4 kV and beam current of 7 mA were applied, using the following sputtering steps: 2 x 2 min, 2 x 5 min, 2 x 15 min. *unknown species.

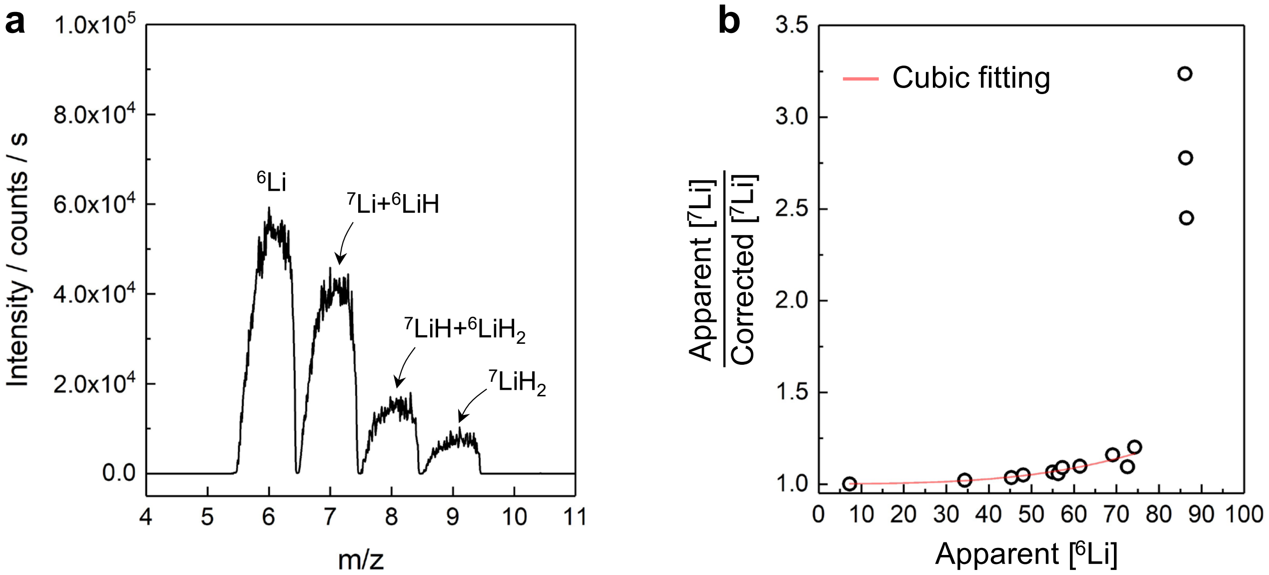

Figure S6. a) PFIB-SIMS positive ion mass spectrum (m/z is mass to charge ratio) measured on the surface of a $^6Li/^7Li$ isotope heterostructure after 60 minutes from the start of the thermal evaporation of ^6Li. A Xe^+ beam with 30 kV acceleration voltage and 4 nA current was directed on a 100 x 100 µm area of the sample. b) Correction factor applied to the diffusivity data to take into account the overlap of ^6LiH and ^7Li signals.

References

1. Crank, J. *The Mathematics of Diffusion*. (Oxford University Press, 1975).
2. Whipple, R. T. P. CXXXVIII. Concentration contours in grain boundary diffusion. *The London, Edinburgh, and Dublin Philosophical Magazine and Journal of Science* **45**, 1225–1236 (1954).
3. Huo, H. *et al.* In-situ formed Li2CO3-free garnet/Li interface by rapid acid treatment for dendrite-free solid-state batteries. *Nano Energy* **61**, 119–125 (2019).
4. Sharafi, A., Meyer, H. M., Nanda, J., Wolfenstine, J. & Sakamoto, J. Characterizing the Li-Li7La3Zr2O12 interface stability and kinetics as a function of temperature and current density. *Journal of Power Sources* **302**, 135–139 (2016).
